# Supplementary material for: Questioning the lasting effect of galvanic vestibular stimulation on postural control
Source: PLoS One. 2019 Nov 7;14(11):e0224619. doi: 10.1371/journal.pone.0224619 (PMC6837330; doi:10.1371/journal.pone.0224619)
Supplement: Attachment — Submitted filename: Response-to-reviewer.docx [file pone.0224619.s002.docx]

Montreal, 1 October 2019

Dr. Nicolás Pérez-Fernández

Academic Editor

PLOS ONE

**PONE-D-19-25269** – “Questioning the lasting effect of galvanic vestibular stimulation on postural control”

Dear Dr. Pérez-Fernández

We would like to thank you and the reviewers for the insightful comments that have been helpful towards significantly improving the manuscript. You will find below the detailed description of the changes we have made.

**REVIEWER #2**

**COMMENT 1:** “Galvanic vestibular stimulation (GVS) is a technique used to stimulate the vestibular system by applying an imperceptible level of electrical current through electrodes placed over the mastoids.” No, GVS can be perfectly perceptible, it depends of the intensity. Please correct. Please mention that GVS stimulate all vestibular sensors and the nerve. You should also quote adequate reviews on GVS in in this first paragraph.

**Author’s response:** We thank the reviewer for his/her vigilance. The passage has been modified as suggested. Indeed, GVS can be perceptible at higher current level. We changed the following sentence to not mention that it is imperceptible. We also added that GVS stimulates the vestibular ends organs and the nerve and included a reference to the only review in this domain.

“Galvanic vestibular stimulation (GVS) is a technique to stimulate the vestibular end organs and nerve by applying a low electrical current through electrodes placed over the mastoids (for a review see [1]).”

**COMMENT 2:** “Postural enhancement” please be more precise.

**Author’s response:** We clarified that point:

“The enhancement of postural control was operationalized and measured as a reduction of “Center of Pressure (CoP) parameters, such as sway velocity and path length.”

**COMMENT 3: “**Might be caused by a learning effect.” Why do you exclude a placebo effect at that point?”

**Author’s response:** We would like to thank the reviewer for this comment. We agree with this suggestion; it could have been a learning effect and/or placebo effect.

“Their experiment, however, did not include a control condition, and it is	therefore possible that the enhancement reported was caused by a learning	and/or a placebo effect.”

**COMMENT 4:** Please give the IRB number, the exact reference of the ethical panel and the date of approval.

**Author’s response:** We added those information:

“Research approval was obtained the 19th February 2018 from the Institutional	Review Board of the Faculty of Medicine at the Université de Montréal (Comité	d’éthique de la recherche en santé; IRB number: 17-178-CERES-D).”

**COMMENT 5:** Why you choose 60 seconds?

**Author’s response:** Previous studies demonstrated that CoP sway measurement of 60 seconds increases test-retest reliability. Because of the repetition of the postural measure (three trials) at each measurement time point, we wanted to make sure that our CoP sway results were reliable. To clarify this point, we explained our choice as followed:

“Three runs of 60 seconds CoP sway measurement were performed at three	different measurement time points: i) prior to stimulation (baseline), ii)	immediately after the end of stimulation (T0: 0h post-stimulation) and iii) one-	hour post-stimulation (T1: 1h post-stimulation). Previous studies have demonstrated that measuring CoP sway for 60 seconds increases test-retest reliability (Rugelj et al., 2015).

**COMMENT 6:** Why choose a foam?

**Author’s response:** CoP sway was measured with a foam surface to reduce somatosensory inputs and subjects had to keep their eyes closed during trials to eliminate the visual input. This procedure has previously been demonstrated to primarily measure the influence of the vestibular system on postural control (Cohen et al., 1993). Here, we stimulated the vestibular end organs and nerve with nGVS and we were interested to specifically examine the influence of such stimulation on vestibular function. To explicitly justify the selection of this postural condition, we modified the following sentences:

“Participants had to perform a static postural control assessment with their eyes closed, standing barefoot on a foam surface (AIB Balance Foam, AIB, USA) placed on a force plateform (Accusway, AMTI, USA). This procedure, with eyes closed on a foam surface was selected as it allows to specifically measure the influence of vestibular inputs on postural control (Cohen et al.,1993).”

**COMMENT 7:** Discuss CoP measurement one-hour post stimulation.

**Author’s response:** We would like to thank the reviewer for this comment. We agree that this information is important to understand the method and we added an explanation of this measurement time point to the text. Several CoP measurement time points could have been performed following the end of stimulation, such as 2 or 3 hours post-stimulation. However, as our objective was not to determine the full duration of the effect of nGVS) we preferred not to perform multiple task repetitions as this could induce a fatigue effect or increase learning effects.

“CoP sway was measured at the end of stimulation to examine the immediate	effect of nGVS on postural control, and one-hour post-stimulation to assess	the sustained effect of nGVS on postural control.”

**COMMENT 8:** Why the subjects were stimulated in a seated position and their posture tested in a standing position?

**Author’s response:** Since we were investigating the effect of nGVS post-stimulation, the position during the stimulation was not taken into consideration. We made sure that all participants had a similar activity/position during the stimulation period in order to reduce the influence of activity on the post-stimulation effect. Furthermore, due to the duration of nGVS stimulation (30 minutes) a standing position could induce a fatigue effect, while a seated position reduces the occurrence of such effect. Interestingly, previous studies examining the post-stimulation effect (Fujimoto et al., 2016; Fujimoto et al., 2018) did not report the position of participants during stimulation.

**COMMENT 9:** Were the subject barefoot?

**Author’s response:** Indeed, subjects were barefoot. This important detail is now mentioned in the manuscript:

“Participants had to perform a static postural control assessment with their eyes closed, standing barefoot on a foam surface (AIB Balance Foam, AIB, USA) placed on a force plateform (Accusway, AMTI, USA). This procedure, with eyes closed on a foam surface was selected as it allows to specifically measure the influence of vestibular inputs on postural control (Cohen et al.,1993).”

**COMMENT 10:** “Therefore, it was not possible for the participant to determine if they received the sham or the real stimulation.” How did you check that?

**Author’s response:** Prior to the study, we performed pre-tests to assess whether there was a difference between the sensation perceived with nGVS and the sham stimulation. Participants were not able to discriminate nGVS and sham. Indeed, due to the low nGVS intensity, a tingling sensation can only be perceived during the ramp up of the current and, to create a similar sensation with the sham stimulation, the current is ramped up for a few seconds before ramped down (for more details, see nGVS section). We modified the following sentence to make it clearer:

“The sham stimulation consisted of the current being ramped up to 1 mA for 30 seconds and then ramped down. This was done to create the same tingling sensation that can be perceived only during the ramp up of nGVS simulation and to make sham trials undistinguishable for nGVS trials.”

**COMMENT 11:** Why is it certain that decreasing postural sway in an “improvement of stability”, at the very least in healthy subjects? It could be also an incidental effect in patients and why it is so sure that it is beneficial? Postural sway could be useful in many ways: exploring the limits of the polygon of sustentation, decreasing the risk that the plantar sol receptors become desensitized etc. Please delete that terminology, which is biased in the discussion and discuss these points. For instance, it could be some sort of freezing reaction, which explains why it was observed in the nGVS group.

**Author’s response:** We would like to thank the reviewer for this comment. As previously demonstrated, a decrease of postural sway parameters was associated with an improvement of postural stability. Sway velocity and path length were demonstrated to be predictors of falls, and therefore an increase in the value of those parameters is related to an increase risk of falls (Tharpa et al., 1996; Piirtola et al., 2006). Furthermore, the reduction of CoP sway parameters observed in our study could be associated to an improvement of postural stability even in healthy subjects, since the postural condition (foam surface and eyes closed) is unusual and difficult even for healthy subjects.

**COMMENT 12:** In the conclusion, one should insist also of comparing the effect of GVS with sham procedures in vestibular patients.

**Author’s response:** We agree with the reviewer’s comment. We added the following sentence to underline the necessity of a sham stimulation with vestibular patients.

“In summary, the present study highlighted the necessity of incorporating a sham condition in the experimental design when investigating the effect of nGVS on postural stability, and more specifically with subjects presenting vestibular dysfunctions. The incorporation of a sham stimulation enables to dissociate the effect of stimulation from possible experimental bias.”
