# Supplementary material for: Questioning the lasting effect of galvanic vestibular stimulation on postural control
Source: PLoS One. 2019 Nov 7;14(11):e0224619. doi: 10.1371/journal.pone.0224619 (PMC6837330; doi:10.1371/journal.pone.0224619)
Supplement: Attachment — Submitted filename: Nooristani_Reply-round2.docx [file pone.0224619.s003.docx]

Montreal, 1 October 2019

Dr. Nicolás Pérez-Fernández

Academic Editor

PLOS ONE

**PONE-D-19-25269** – “Questioning the lasting effect of galvanic vestibular stimulation on postural control”

Dear Dr. Pérez-Fernández

We would like to thank you and the reviewers for the insightful comments that have been helpful towards significantly improving the manuscript. You will find below the detailed description of the changes we have made.

**EDITOR COMMENTS :** Still comments 8 and 11 from Reviewer 2 must appear on the final text.

**COMMENT 8:** Why the subjects were stimulated in a seated position and their	posture tested in a standing position?

**COMMENT 11:** Why is it certain that decreasing postural sway in an	“improvement of stability”, at the very least in healthy subjects? It could be also	an incidental effect in patients and why it is so sure that it is beneficial? Postural	sway could be useful in many ways: exploring the limits of the polygon of	sustentation, decreasing the risk that the plantar sol receptors become desensitized	etc. Please delete that terminology, which is biased in the discussion and discuss	these points. For instance, it could be some sort of freezing reaction, which	explains why it was observed in the nGVS group.

**REVIEWER #2 COMMENT 1:** Please explain in the text and not only to me: why the subjects were stimulated in a seated position and their posture tested in a standing position?

**Author’s response:** We added those information:

“The white noise ranged between 0 to 640 Hz and stimulation was applied continuously for 30 minutes. All subjects were sitting during the stimulation period to reduce the influence of an ongoing activity during the stimulation on the effect of nGVS post-stimulation. Furthermore, considering the duration of nGVS stimulation, the seated position prevents a fatigue effect that could occur during a standing position or during other position.”

**REVIEWER #2 COMMENT 1:** Why is it certain that decreasing postural sway in an “improvement of stability”, at the very least in healthy subjects?

**Author’s response:** We added that information in the introduction:

“Indeed, an increase in the value of sway velocity and path length is related to an	increase risk of falls (Tharpa et al., 1996; Piirtola et al., 2006), therefore a reduction	of those parameters is considered as an improvement of postural control”
